# Supplementary material for: Are carbon emissions trading and green financial instruments synergistic? -Comprehensive quantitative research based on content analysis
Source: PLoS One. 2024 Mar 7;19(3):e0298601. doi: 10.1371/journal.pone.0298601 (PMC10919601; doi:10.1371/journal.pone.0298601)
Supplement: S1 Data — (ZIP) [file pone.0298601.s001.zip › Data/地级市Z_F工作报告.zip!/╔╜╢1⁄2/╢1⁄2╙¬╩╨/└┤╫╘╣1⁄2╓┌║┼í╛┬φ┐╦╩2╛▌═°í┐_2010.docx]

各位代表：

　　现在，我代表市人民政府向大会报告政府工作，请予审议，并请市政协委员和其他列席会议的同志提出意见。

一、2009年政府工作回顾

　　过去的一年，是挑战严峻、极不平凡的一年，也是我们迎难而进、成效明显的一年。一年来，在党中央、国务院，省委、省政府和市委的正确领导下，全市上下认真贯彻党的十七大和十七届三中、四中全会精神，团结拼搏，改革创新，攻坚克难，黄河三角洲高效生态经济区开发建设顺利推进，上升为国家战略；经济社会发展逆势而上，各项指标平稳较快增长。初步统计，2009年全市实现生产总值2076.6亿元，比上年增长12.4%；规模以上工业增加值1457.9亿元，增长13.1%；全社会固定资产投资1095.6亿元，增长23.4%；社会消费品零售总额303.3亿元，增长19.1%；地方财政收入80.87亿元，增长13.98%；金融机构各项存款余额1301亿元，比年初增加268.2亿元，贷款余额899.4亿元，比年初增加203.5亿元；万元生产总值能耗、二氧化硫排放量、化学需氧量排放量均实现了控制目标。

　　(一)全力打好“三大战役”，保持经济平稳较快发展。去年初，金融危机席卷全球，原油价格急剧下滑，企业经营举步维艰，经济严冬骤然降临。面对突如其来的严峻挑战，全市上下团结一致，沉着应对，共克时艰，全力打好扩大投资突破战、企业运行保卫战、结构调整攻坚战，一季度站稳了脚跟，二季度趋于平稳，下半年整个经济呈现出回升向好的局面。在扩大投资方面，狠抓大项目建设，共开工投资5000万元以上项目167个，总投资476.5亿元；积极争取中央和省投资项目，及时落实配套资金。强有力的投入，有效遏止了经济下滑态势，固定资产投资拉动经济增长8个百分点。在保企业运行方面，年初，面对复杂多变的经济形势，我们迅速出台了14条政策措施，成立了工业经济运行指挥部。先后与省中行、省农行签订了战略合作协议，召开了3次政银企合作洽谈会，签约金额515亿元，市财政安排4000万元设立了中小企业互助资金，成立了4家小额贷款公司，有效缓解了企业资金困难。进入下半年，面对企业经营持续困难，又制定出台了30条政策措施，实行了市级领导和市直部门帮扶企业制度，先后梳理并解决了企业急需解决的28个问题。由于我们主动超前，措施有力，全市企业不但没有出现大面积停产现象，而且规模以上工业企业增加了118户，亏损企业减少了22.7%，地方规模以上工业增加值增长23.2%、利税增长27.7%，地方工业在全市工业中的比重由上年的46.3%提高到64.6%。在结构调整方面，充分运用国际金融危机形成的倒逼机制，加快结构调整步伐。制定出台了工业调整振兴意见和六大产业发展规划，实行一个产业、一个协会、一个融资平台、一个展销平台，加快构筑特色产业体系。新兴产业初具规模，电子信息、汽车及零部件、新能源等产业快速发展，新上了一批科技含量高、带动力强的大项目。全市高新技术产业产值占工业总产值的比重达到22.2%，比年初提高3个百分点。传统产业得到优化提升，开展了石油装备制造业整合试点工作，成功举办了2009中国(东营)国际石油石化装备与技术展览会；橡胶轮胎业，依托行业协会，在采购、研发、销售等环节加快整合；石油化工业，深入推进与中海油的合作，成功举办了2009中国化工园区发展论坛。突出发展以文化旅游业为重点的服务业，重点景区建设加快推进，成功举办了第二届黄河文化旅游博览会，发起成立了沿黄城市旅游促进会。现代物流、科技信息等生产性服务业快速发展，商贸餐饮等生活性服务业进一步提升。服务业增加值增长12.2%，占生产总值的比重提高2.1个百分点。统筹区域经济发展，市属与县域协调推进，市属经济规模不断壮大，县域经济实力进一步增强。

　　(二)加快黄河三角洲开发建设，推动高效生态经济区上升为国家战略。去年，尽管经济运行压力巨大，但是我们坚持黄河三角洲开发建设总战略不动摇，一手抓经济健康运行，一手抓黄河三角洲开发，推进工作实现重大突破。3月份，国家部委联合调研组到东营对高效生态经济区进行了专题调研。之后，我们组成7个专业小组，由市级分管领导和部门主要负责同志负责，盯上靠上与国家部委对接，全力做好规划编制跟进工作。11月23日，国务院批复了黄河三角洲高效生态经济区发展规划，标志着黄河三角洲开发正式上升为国家战略。我们正在做的工作完全符合国家规划要求，为加快实施国家战略打下了良好基础。“四区一城”主体产业区建设取得重大进展。临港产业区，中海油物流园、大唐东营发电项目积极推进，园区基础设施配套建设得到加强，一批项目开工建设，9个项目已经投产，东营港经济开发区被列为中国石油化工产业区。高端产业区，生态工业园、软件园、“生态谷”、新能源产品及装备制造基地建设快速推进，引进建设了新型功率半导体、泰克拓普等8个电子信息项目，吉奥、俊山五菱、东方曼3个整车制造及一批汽车零部件项目，风电法兰等11个新能源项目，新签约过亿元项目28个，高端产业正在加快聚集。黄河口生态旅游区，总体规划获得国家批复，游客综合服务中心主体完工，游船码头投入使用，黄河口湿地公园正在建设。现代渔业示范区，4.1万亩起步区基础工程全面完成，3万亩养殖区投入生产，研发中心大楼主体完工。黄河水城，广利河三年治理工程全面完成，“三园一区”形成景观，体育公园竣工；文化公园广场全面完工，国际会展中心投入使用；科技公园软件大厦建成投用；清风湖景区主体完工；胜利大街桥、东二路桥、东三路桥建成通车。“三网”绿化工程取得明显成效，三年累计绿化面积45.5万亩，基本构建起了“三网”绿化大框架。立体交通体系建设加快推进，德大铁路开工建设；东营港实现开港通航，散杂货泊位投入运营；机场飞行区和航站楼主体完工。

　　(三)大力发展现代农业，农业结构调整取得新成效。粮棉喜获丰收；渔业、畜牧等优势产业快速发展，特色水产品养殖面积达到161万亩，黄河口大闸蟹入选中国十大名蟹，效益大幅提高；引进和培育了一批畜牧龙头企业，促进了规模化养殖，人均畜产品生产能力、畜牧产值均居全省首位；休闲观光农业发展势头良好，重点园区建设取得明显成效。着力强化农产品加工销售环节，扶持发展农业龙头企业和农民专业合作组织，提高了农业产业化经营水平。加强农业农村基础设施建设，疏浚治理灌渠4条、排河6条，扩建加固水库4座，维修改造农村危桥44座，新建改造农村公路393公里。着力改善农村人居环境，新建农房20934户，改造危房5359户，解决了8.4万农村人口饮水安全问题，新增沼气用户9800户，农村面貌有了新改观。加大对“三农”的投入，强农惠农政策全面落实。

　　(四)不断深化改革开放，发展活力明显增强。发展高效生态经济改革试点前期工作顺利推进。投融资改革取得新成效，“四区一城”融资平台运行机制不断完善，市场化运作水平进一步提高；引进股份制银行工作实现突破，恒丰银行、华夏银行落户东营。国有土地清理整顿深入开展。林权制度改革试点进展顺利。企业改革不断深化，10家企业进入上市程序。政府机构改革启动，事业单位综合配套改革稳步推进。医药卫生体制改革全面展开。努力稳定外需，及时出台了促进外经贸发展的政策措施，积极帮助企业应对贸易摩擦，实现进出口总值39.6亿美元，其中出口17.6亿美元。大力开展产业招商、专业招商，举办了6次高层次境内外招商活动，吸收外商直接投资1.65亿美元。

　　(五)加大保障民生力度，人民生活水平进一步提高。千方百计增加城乡居民收入，城镇居民人均可支配收入21313元，增长9.4%，农民人均纯收入7327元，增长10%。加强就业再就业工作，统筹抓好各类群体特别是高校毕业生和就业困难人员就业，新增城镇就业4.1万人，农村劳动力转移就业5.97万人，城镇登记失业率为1.87%。完善社会保障救助体系，在校大学生全部纳入城镇居民医疗保险范围，提高了农民医疗保险财政补助标准和企业养老保险、失业保险金发放标准。黄河南展区房台拓展工程全部完成。建设了一批保障性住房，实行了经济适用房和廉租房货币化补贴，启动了中心城棚户区改造工程，积极支持油田职工住房建设，改善了群众住房条件。加大食品药品安全整治力度，保障了群众饮食用药安全。采取有力措施，及时加强甲型H1N1流感、手足口病防控。改善城市居民饮用水水质等10件便民实事全部完成。扎实做好援川工作，完成投资1.3亿元，坝底中学、卫生院等7个项目竣工使用。

　　(六)扎实推进社会建设，和谐稳定局面进一步巩固。各级各类教育协调发展，教育质量进一步提高。优化教育布局，中心城区3所小学开工建设，市技术学院和胜利一中迁建进展顺利。加大公共卫生投入，市人民医院病房楼建成投用，实施乡镇卫生院、城市社区卫生服务站、农村卫生室建设等工程，实行城市社区100种常用药品“零差率”销售，有效缓解了看病难、看病贵问题。科技、信息事业加快发展，我市被列为国家知识产权工作示范城市和全国农村信息化试点城市。社会主义核心价值体系建设扎实推进，“从我做起、走向文明”主题实践活动全面展开。积极推动文化事业和文化产业发展，一批城乡文化设施建成投用，市艺术团改企转制稳步推进。新闻出版、广播影视、文学艺术、社会科学等事业健康发展。全民健身活动广泛开展，成功举办了黄河口国际马拉松赛，圆满完成了第十一届全运会赛事承办工作。认真抓好人口和计划生育工作，全市人口自然增长率为3.3‰。国防教育、人民防空、民兵预备役、双拥共建和优抚安置工作成效明显，妇女儿童、青少年、残疾人、慈善、老龄事业持续发展，外事侨务、民族宗教、对台、档案、统计、史志、无线电管理、仲裁、气象、防灾减灾和红十字会等工作取得新成绩。

切实加强民主法制建设，普法和依法治理工作深入开展。规范行政审批工作，建成了行政审批电子监察系统。畅通社情民意渠道，开通了市长公开电话，建立了市长市民对话会制度。加强信访工作，认真解决群众反映的问题。大力推进平安东营建设，全面深化社会治安综合治理，扎实推进“打黑除恶”等专项行动，圆满完成了新中国60周年大庆和第十一届全运会安保任务，维护了社会稳定。毫不放松地抓好安全生产，加大隐患排查整治力度，保持了安全生产形势持续稳定。完善应急管理体系，突发事件应急处置能力进一步提高。

　　各位代表！回顾2009年，我们充满自豪，充满自信。这一年，是东营发展历史上具有里程碑意义的一年。黄河三角洲开发上升为国家战略，我们梦寐以求的夙愿得以实现，东营吸引了世界的目光。这一年，胡锦涛总书记亲临东营视察，全市上下群情振奋，倍受鼓舞，凝聚起继往开来、无往不胜的巨大力量。这一年，我们经历了国际金融风暴和油价大幅下滑的严重袭击，不仅没有被困难击倒，而且在风雨中展开搏击的翅膀，书写了东营发展史上腾飞崛起的新篇章。成绩来之不易，经验弥足珍贵。在巨大的困难和考验面前，我们的企业特别是企业家，相信党委政府，勇担社会责任，敢于迎难而上，成为抗击金融风暴的中流砥柱。我们的干部特别是广大基层干部，牢记历史使命，勇于攻坚克难，积极化解矛盾，成为支撑东营发展稳定的坚强栋梁。社会各界和广大人民群众，团结包容，同舟共济，维护大局，成为推动东营持续发展的力量源泉。油地军校、中央和省驻东营各单位，通力合作，相互支持，形成了抗击风暴、战胜困难的强大合力。在此，我代表市人民政府，向全市人民，向各民主党派、工商联、各人民团体和各界人士，向驻东营人民解放军、武警官兵和中央、省驻东营单位，向所有为东营的建设和发展作出贡献的同志们，致以崇高的敬意，表示衷心的感谢！

　　在充分肯定成绩的同时，我们也清醒地认识到经济社会发展面临的矛盾和问题。主要是，经济发展方式粗放，经济结构不合理，服务业发展滞后，自主创新能力不强，节能减排任务艰巨，转方式调结构压力大、任务重；部分企业特别是中小企业生产经营仍很困难，市场低迷、企业效益不高的局面还没有根本改观；财政增支减收因素明显增多，收支平衡压力进一步加大；城乡居民收入差距较大，农民持续增收困难；保障改善民生的长效机制尚不健全，一些民生问题还没有解决好。政府工作与形势的要求和人民的期望还有不小差距，群众对干部作风和政府效率不够满意。对这些问题和困难，我们一定要高度重视，采取扎实有效的措施，努力加以解决。

　　各位代表！展望2010年，前景光明，充满希望。今天的东营，已经站在一个蓄势待发、喷薄欲出、世人注目的新的历史平台上。但是，前进的道路上，还会遇到许多可以预料和难以预料的艰难险阻，可谓雄关漫道，任重道远。艰难困苦，玉汝于成。只要我们全市上下、油地军校、社会各界团结包容，万众一心，在攀登的道路上坚韧不拔、永不言退，在困难挫折面前百折不挠、永不言败，我们就一定能抓住难得的历史机遇，在黄河三角洲这片年轻的土地上，创造新的辉煌，书写我们这一代人的光荣与梦想！

二、2010年政府工作总体要求

　　今年是实施国家战略的第一年，也是实施“十一五”规划的最后一年。做好今年的工作，对于加快黄河三角洲开发建设步伐，保持经济平稳较快发展，为“十二五”规划启动实施奠定良好基础，具有十分重要的意义。

　　今年政府工作的总体要求是：全面贯彻党的十七大和十七届三中、四中全会精神，深入贯彻落实科学发展观，深入贯彻落实胡锦涛总书记视察东营时的重要讲话重要指示精神，全力实施国务院批复的黄河三角洲高效生态经济区发展规划，按照“新起点新阶段新目标新局面”的工作要求，把握抢抓机遇、积极作为的工作基调，着力抓好高效生态经济区建设、转变发展方式调整经济结构、保障改善民生三项战略任务，努力开创经济加快发展、社会全面进步、黄河三角洲开发快速推进的新局面。

　　今年经济社会发展的主要预期目标是：全市生产总值增长11%，全社会固定资产投资增长23%，社会消费品零售总额增长17%，进出口总值增长10%，吸收外商直接投资增长10%，地方财政收入增长11%，城镇居民人均可支配收入增长8%，农民人均纯收入增长9%，城镇登记失业率控制在2.4%以内，居民消费价格总水平涨幅控制在3%以内。主要约束性指标是：万元生产总值能耗降低4.41%，二氧化硫排放量削减13%，化学需氧量排放量削减4%，人口自然增长率控制在5.9‰以内。

　　实现今年经济社会发展的目标，必须把握好“五个着力”的基本要求：着力推动可持续发展，把保持经济平稳较快发展与结构调整结合起来，在巩固经济回升向好势头的同时，进一步加快转变发展方式，调整经济结构，大力推进经济社会生态化。着力推动均衡发展，加大投资力度，努力扩大消费，积极开拓国际市场。着力推动一体化发展，围绕高效生态经济区发展规划，统筹空间布局和产业发展，把实施重点区域带动战略与培植产业结合起来，集中突破主体产业区；把统筹城乡协调发展与推进城镇化结合起来，努力形成新型城镇化、新型工业化、农业现代化良性互动的局面。着力推动创新发展，加大自主创新力度，努力在关键技术研发和人才培养引进方面取得突破；抓好重点领域和关键环节的改革，创新体制机制，增强经济发展的内在动力和活力。着力推动协调发展，更加注重保障改善民生，更加注重发展社会事业，促进社会和谐。我们务必科学判断形势，准确把握大局，奋力抢抓机遇，乘势开拓前进，努力夺取经济社会发展的新胜利！

三、实施国家规划加快推进黄河三角洲开发建设

　　今年，我们必须毫不动摇地坚持黄河三角洲开发这个总战略，举全市之力，集全民之智，高点定位，深度推进，迅速拉开大开发大开放的新格局。在工作中，要突出“一个特色”，坚持“两个推进”，强化“三个保障”。

　　突出“一个特色”，就是坚定不移地突出高效生态的特色，大力推进经济社会生态化建设。一是着力发展生态化产业。转变经济发展模式，以生态化标准改造提升传统产业，加快培植新信息、新能源、新材料等战略性新兴产业，发展循环经济和低碳经济，加快构建高效生态产业体系。加大节能降耗力度，加快淘汰落后产能。积极推广循环生产模式和清洁生产技术，建设一批生态工业示范园区，实现绿色增长。二是着力建设生态化城市。加快打造水城景观，以广利河治理为重点，贯通城区水系，形成多条循环水路。继续推进“三网”绿化工程，高标准实施广利河两岸绿化，建设一批高效林业示范区，实现市内交通干线绿化全线贯通，确保完成绿化面积26.3万亩。加大污染治理力度，坚决打好小清河、广利河治污战役。制定计划，出台政策，加快城边化工企业搬迁。年内建设4座污水处理厂、2座垃圾无害化处理场，搞好中心城雨污分流管网改造。实施自然保护区湿地生态修复工程、黄河清八治理工程、刁口河流路生态补水工程，改善黄河口生态环境。三是着力构建生态化社会。把生态建设体现到社会发展的方方面面，让人民群众共享生态文明成果。搞好农村面源污染治理，改善农村生态环境。大力推进建筑节能，实施老居住区供热分户计量改造，推进太阳能与建筑一体化工程，大力发展地源热泵技术。积极推广节水新技术，发展中水回用和再生水利用。鼓励使用LED等节能灯具，积极发展电动公交车，引导公众绿色消费。四是大力弘扬生态文化。牢固树立生态理念，加强对生态文化的研究和挖掘，搞好生态文化资源的保护与开发，发展具有东营特色的生态文化产业，打造生态文化品牌。广泛开展生态文明宣传教育，普及生态科学知识，努力在全社会形成保护生态环境的价值观念、生活方式和消费行为。

　　坚持“两个推进”，就是推进产业发展和城镇化建设。

　　以四大主体产业区为主要载体，迅速形成产业聚集发展的格局。临港产业区，要加快中海油原油储备基地、成品油仓储区建设，做好炼化一体化项目前期工作；加快推进大唐东营发电项目，争取尽快开工建设；制定有力措施，推动化工产业向港区集中；加大投入力度，加快基础设施配套建设，增强承载能力。高端产业区，要以大项目为支撑，以产业链为基础，着力打造高端产业集群，突出抓好生态工业园、软件园、新能源产品及装备制造基地建设；按照建设新城区的要求，调整完善总体规划，重点推进“一轴、三片”开发，拉开一片新城区。黄河口生态旅游区，要加快推进游客综合服务中心和湿地公园建设，力争上半年投用；规划建设汽车野营地等一批旅游项目，适时启动黄河口生态休闲旅游度假区建设；理顺管理体制，提高运营管理水平。生态高效农业区，要抓紧启动现代渔业区二期工程，完成研发中心、商务中心及配套设施建设；编制渔业区小城镇规划并启动实施；创新模式，组织本地农民进区经营；规划建设现代畜牧业开发区，争取一批龙头企业进区建设。

　　以打造黄河水城为龙头，大力推进新型城镇化。按照中心城、县城与卫星城镇、小城镇、农村社区四个层级，高起点修编城镇体系总体规划，加快配套完善各类详细规划和专业规划，形成空间布局合理、服务功能健全、城乡一体化发展的城镇体系。加强规划管理，实行城乡规划一体化审批制度，严格审批程序，维护城镇规划的严肃性。突出做强做优中心城，按照“东扩、西疏、南展、北延、中优”的思路，规划建设新城区，拉开中心城发展新格局。重点搞好广利河沿岸深度开发，全力打造黄河水城精华区，形成旅游文化景观长廊和服务业经济隆起带。今年重点推进汽车总站、文化公园、清风湖风景区、奥体中心等片区的开发建设，开工建设会展中心大酒店和写字楼、黄河文化博物馆等项目。加强城市供水、供热、供气、公交等设施建设，实施南三路、胜利大街南延、东三路北延等道路建设。加强城市环境综合整治，推进济南路和城中村改造，搞好绿化、美化、亮化，全面提升城市环境。加强县城规划建设管理，完善基础设施和公共服务设施，增强综合承载能力，加快建设现代化的中小城市。大力培育中心镇，提高其带动周边农村发展的能力。改善农民工就业、居住、医疗、子女就学等基本生活条件，逐步放宽城镇户籍限制，促进有条件的农民工有序转为城镇居民。结合新农村建设，推进城镇基础设施和公共服务向农村延伸，建设新型农村社区，促进农民居住社区化、农民生活城市化。

　　强化“三个保障”，就是着力在深化改革、扩大开放和基础设施建设上突破，为高效生态经济区建设提供强力支撑。

　　一是深化重点领域和关键环节的改革。第一，全面启动高效生态经济改革试点。统筹安排，分步推进，加快建立有利于高效生态经济区发展和资源型城市转型的体制机制。第二，大力推进金融创新。以推动直接融资为重点，加快金融创新步伐，切实提高金融服务和保障能力。加快组建黄河三角洲创业投资集团，推动设立黄河三角洲发展银行、产业投资基金和产权交易中心，积极争取发行政府债券，支持发行企业债券，争取设立村镇银行，大力发展融资担保机构，吸引国内外金融机构来东营设立分支机构。鼓励银银、银保合作，拓展融资空间。第三，全面启动土地管理制度改革。开展未利用地开发管理改革试点，重点在未利用地开发利用方面先行先试，积极探索土地利用规划动态管理模式，实行土地利用计划差别化管理，稳步推进城乡建设用地增减挂钩试点。第四，稳妥推进农村各项改革。坚持农村基本经营制度，落实好农村土地承包政策，探索建立农村土地流转机制。搞好农村产权和集体林权制度改革。加快推进农场改革，建设新型农业经济区。第五，积极推进公用事业改革。按照政府主导、市场化运作的思路，逐步推进城市供水、供热、供气、公交、污水和垃圾处理等公用事业改革，理顺价格关系，加快建立投资多元化、运行市场化、管理规范化的新机制。第六，加快推进城市管理体制改革。按照“分层次、一体化”的原则，创新管理机制，构建大城管格局，提高城市管理水平。第七，深化企业改革。支持企业建立完善现代企业制度，推进企业兼并重组，支持优质企业上市。放宽市场准入，增强非公有制经济和中小企业发展活力。

　　二是进一步扩大对外开放。坚持把黄河三角洲开发置于世界经济发展的大格局中去审视、去把握，以开放的视野和举措汇聚各方资源、凝聚各方力量，以大开放促进大开发、大发展。要打好国家战略牌，大力开展产业招商、专业招商。重点围绕“四区一城”和产业发展规划，瞄准境内外重点地区集中开展招商活动，加强与世界500强、国有大企业和行业领军企业的战略合作，加快引进一批“大高外”项目。切实抓好签约项目的推进落实，增强招商引资实效。办好黄洽会等大型经贸活动。加强对外交流合作，重点搞好与周边地区特别是天津滨海新区、辽宁沿海经济带等地区的经济交流和技术合作。

　　三是加快完善基础设施。重点加快东营港建设，依托中海油大力推进2个5万吨和2个5千吨液体化工泊位建设，加快散杂货泊位改造，积极推进港口二期工程规划建设，动员社会力量建设业主码头。进一步完善广利港规划，尽快开工建设。全面完成飞机场改造任务，升级为4D级，增开航班航线。积极推进黄大铁路、德大铁路、飞机场至自然保护区公路建设，争取年内全线开工。积极推动环渤海高等级公路、东营至滨州高速公路以及东营港疏港铁路等铁路支线规划建设。加快完善防潮体系，重点搞好东营港区段和东营经济开发区临海新堤建设，维修加固北部防潮堤。加强能源建设，实施一批火电风电项目和输变电工程，完善电网设施。深入推进“数字化东营”建设，提高信息化水平。

四、努力保持经济平稳较快发展

　　保持经济平稳较快发展，是今年政府工作的重要任务。必须用好国家宏观经济政策，强化投资消费出口的拉动作用、企业的支撑作用、财税的保障作用，努力保持较快的经济增长速度。

　　(一)加大投资保增长。抓住国家宏观经济政策相对宽松的机遇，继续加大投资力度，确保全年固定资产投资突破1300亿元。加强重点项目建设，加快推进180个已开工项目，确保按期建成投产，实施好346个投资1000万元以上的新开工项目。突出投资重点，围绕高效生态经济区建设，加快实施一批重大基础设施项目、高新技术项目和现代服务业项目。进一步做好中央和省扩大内需资金争取工作，突出抓好机场、港口、防潮堤等项目资金的争取。把握好投资方向，优化投资结构，防止低水平重复建设。鼓励和扩大民间投资，引导支持民间投资进入基础产业、基础设施、市政公用事业、社会事业、金融服务等领域。加强项目论证和储备，为加大投资提供支撑。规范项目运作，严格资金监管，切实提高投资质量和效益。

　　(二)扩大消费保增长。千方百计增加城乡居民收入，提高居民特别是中低收入群体消费能力。培植消费热点，大力发展社区商业、物业、家政等便民消费，扩大旅游休闲、文化娱乐、体育健身等服务消费，引导和促进汽车、住房等大宗商品消费，推动消费结构优化升级；积极引导农民消费需求，扩大“万村千乡”市场工程和“两进”工程覆盖面，落实“家电下乡”、农机具购置补贴等政策，开拓农村消费市场。改善消费预期，优化消费环境，维护消费者权益。完善政府储备与商业储备体系，提高市场应急调控能力。

　　(三)稳定出口保增长。深入实施市场多元化战略，落实扶持政策，支持企业参加国际知名展会、建立境外自主营销网络，稳定传统市场，开拓新兴市场。帮助企业用好出口信用保险、出口退税、出口信贷等政策，提升外贸企业国际竞争力。加快转变外贸增长方式，调整优化出口商品结构，扩大自主品牌产品出口，大力发展服务贸易。积极扩大先进技术设备、关键零部件和能源原材料进口。加快“走出去”步伐，支持有条件的企业对外投资，拓展发展空间。指导企业妥善应对贸易摩擦。

　　(四)扶持企业保增长。企业是经济之源、发展之本。在当前企业经营仍面临较多困难的情况下，必须继续强化扶持措施，帮助企业走出困境，加快发展。进一步完善领导、部门帮扶企业制度，落实并指导企业用好各类扶持政策，积极为企业救急解难。进一步完善政银企合作机制，引导金融机构多授信、多放贷；用好中小企业互助资金，完善中小企业信用担保体系，发挥小额贷款公司作用，拓宽企业融资渠道。继续实施大企业带动战略，推动龙头企业与国内外大企业开展合资合作，努力培育一批具有国际竞争力的大企业集团。实施中小企业成长计划，筛选一批成长性强的中小企业进行重点扶持。落实税收优惠政策，全面清理和规范各种收费，切实减轻企业负担。引导企业推进管理创新，规范运作，降低成本，提高效益。

　　(五)加强财税保增长。加强财源建设，加大依法治税管费力度，努力保持财政收入稳定增长。积极推进财税体制改革，继续深化部门预算、国库集中收付、政府采购和政府投资评审等制度改革，深化“金财”工程建设，提高财政精细化管理水平。搞好国有资产管理和运营，盘活有效资产，实现保值增值。牢固树立过紧日子的思想，大力压缩一般性支出，集中财力办大事、保重点。进一步强化审计监督，维护良好的财经秩序。完善政府投资体制，加强政府投资项目监管，积极推行代建制。做大“四区一城”融资平台，加快资产整合，创新融资模式，提高融资能力。

五、加快调整经济结构转变发展方式

　　转方式、调结构是中央和全省经济工作会议的要求，是发展高效生态经济的必然选择，是提升我市经济发展水平的根本途径。无论是解近忧还是谋长远，都要求我们必须坚决打好转方式、调结构这场硬仗。

　　(一)大力调整农业结构促进农民增收。坚持农业三次产业联动发展，突出致富农民这个根本，认真落实强农惠农政策，促进农业不断增效，农民持续增收。一是加快发展现代农业。在稳定粮棉生产的同时，大力培植特色优势产业，集中建设一批规模化、标准化生产基地。重点建设三大园区，在加快推进现代渔业示范区建设的同时，规划建设现代畜牧业示范区和现代农业综合示范区。突出发展渔业、畜牧、林业、蔬菜、花卉和休闲观光农业六大产业。扩大黄河口大闸蟹、海参等名优水产品养殖规模，努力将其培育成在全省乃至全国有影响力的产业；加快推进现代畜牧业基地建设，完善提升畜牧养殖园区，新增100个标准化畜禽养殖小区；结合“三网”绿化，大力发展经济林和林木加工业，提高林业产业的质量和效益；积极发展设施蔬菜生产，建设10处蔬菜标准化生产示范基地；加快发展特色花卉种植，鼓励发展花卉交易市场，促进花卉产业做大做强；加快建设15个休闲观光农业示范园区，扩大规模，提升档次。二是着力提高农业产业化水平。集中扶持农业龙头企业发展壮大，力争年销售收入过亿元的龙头企业发展到50家。加大黄河口特优农产品品牌培育和宣传推介力度，健全完善营销网络，积极推动产销对接。实施农民专业合作社示范工程，重点培育50家示范合作社，带动农民参与产业化经营。大力发展农村二三产业，加强农民实用技术培训，增加农民务工、经营和财产性收入。三是加强农业农村基础设施建设。围绕提高农业综合生产能力，突出抓好南水北调、河道治理、灌区节水改造等水利工程建设，改造中低产田10.5万亩。围绕改善农民群众生活条件，建设改造农村公路255公里，新建户用沼气池6000个，继续实施农村饮水安全巩固工程，搞好农村电网续建配套。制定实施黄河南展区经济社会发展规划，加快建设新农村、新社区、新的黄河观光带。

　　(二)加快工业调整振兴步伐。实施高端、高质、高效产业发展战略，认真落实全市工业调整振兴意见和六大产业发展规划，用好重点产业“四个一”推进机制，全面提升产业整体水平和竞争力。一是重点突破高新技术产业和先进制造业。高新技术产业，以电子信息、新材料、生物医药为重点，加快推进大规模集成电路、新型功率半导体等高科技项目建设，抢占产业发展制高点。先进制造业，重点发展汽车及零部件、石油装备、新能源装备三大产业，加快培育一批龙头型、基地型大项目。二是加快改造提升传统产业。支持企业引进世界一流装备，用高新技术和先进适用技术，改造提升石化、纺织、轮胎等传统产业。积极推进产业整合，鼓励优势企业与国内外大企业强强联合，鼓励关联产业、上下游企业联合重组。三是着力发展蓝色产业。坚持海陆统筹，巩固提升渔业、盐业、盐化工等传统产业，积极发展海洋生物医药、海洋装备制造、海水综合利用等先进制造业，发展海上运输、滨海旅游等海洋服务业，培植一批新的增长点。

　　(三)加快发展现代服务业。重点突破文化旅游业，加快建设黄河口生态旅游区、黄河水城河海风情旅游区、孙子文化旅游区三大景区，引进国内外知名旅游公司，搞好市场化运作，形成拳头旅游产品。加强沿黄城市间的合作，共同打造“黄河文明”旅游线。大力发展现代物流业，重点推进东营经济开发区保税物流等一批物流园区、物流中心建设，加快发展第三方物流，培育一批现代物流企业集团。加快发展软件和服务外包业，以东营软件园为依托，推进云计算中心等项目建设，吸引国际知名服务外包企业到东营建立分包中心和承接基地。促进金融、保险、房地产业健康发展。进一步改造提升商贸餐饮等传统服务业，加快发展商务服务、休闲娱乐、社区服务等新兴服务业。

　　(四)加快调整区域经济结构。以东营经济开发区、东营港经济开发区为重点，加快发展市属经济。以县区省级经济开发区为重点，着力打造富有特色的产业集群，促进县域经济加快发展；落实促强扶弱措施，继续实施“突破利津”战略。促进油地军校融合发展，大力支持胜利油田增储上产、加快存续企业改制、改善职工群众生活；大力支持石油大学提高办学水平、转化科技成果；大力支持济军生产基地培育新的经济增长点。

　　(五)努力提高科技创新能力。加快建设中国石油大学国家大学科技园及其“生态谷”，依托黄河三角洲可持续发展研究院，规划建设一批高层次研发机构。支持骨干企业建设一批工程技术研究中心、企业技术中心、重点实验室和院士工作站，提高企业自主研发能力。重点建设国家石油装备工程技术研发中心。加强与国家科研院所、高等院校联合攻关，重点在电子信息、新材料、生物技术等关键领域和核心技术研发上取得突破。深化产学研结合，整合科技创新资源，构建一批产业技术创新战略联盟，加快引进转化一批科技成果。自主创新关键在人才。深入实施人才强市战略，加大人才培养力度，重点培养一批现代产业领军人才和高素质技能型人才，盘活用好现有各类人才；实施“十百千”人才引进计划，吸引一批海内外高层次人才来东营创业；健全有利于科技创新的评价、考核、激励机制，加强知识产权的使用和保护，营造良好的创新创业环境。

六、保障改善民生促进社会和谐稳定

　　坚持把保障改善民生放在更加突出的位置，着力解决人民群众最关心、最直接、最现实的利益问题，不断提高人民生活水平，促进社会和谐稳定。

　　(一)着力解决民生问题。千方百计扩大就业，年内新增城镇就业3万人，农村劳动力转移就业3万人，努力保持就业形势基本稳定。继续实施积极的就业政策，重点做好高校毕业生、零就业家庭、下岗失业和关闭破产企业人员就业再就业工作，加快开发公益性岗位，妥善安置就业困难人员。充分利用国家就业扶持政策延长一年的有利时机，继续做好援企稳岗工作。全面推进创业带动就业，建立30个创业就业培训基地和50个创业孵化基地。加快建立服务、培训、维权三位一体的农村劳动力输出工作机制，引导农民外出就业、就地创业。强化公共就业服务，健全失业动态监测体系，建立统一开放的城乡人力资源市场。完善社会保障救助体系。加快推进城镇职工基本医疗保险制度建设。完善新型农村合作医疗制度，实现市级统筹，总体筹资水平每人每年不低于140元；城镇居民基本医疗保险最高支付限额由6万元提高到10万元。做好社会保险关系转移接续工作，继续提高企业退休人员养老金支付标准。开展新型农村养老保险工作试点。进一步扩大社会保险征缴面，引导非公有制经济从业人员、农民工和灵活就业人员积极参保。扩大保障性住房建设，新建710套廉租房、400套经济适用房、600套农村特困群众安居房，发放廉租房货币化补贴1040户、经济适用房货币化补贴650户。加快实施棚户区改造工程，建设安置房500套。继续支持油田职工住房建设。推进放心粮油食品工程和农户科学储粮工程。积极发展慈善事业，实施一批慈善救助项目。办好10件便民实事，确保按期完成。继续做好援川工作，实施北川新县城道路、民生大楼等建设工程。

　　(二)大力发展教育卫生等社会事业。坚持教育优先发展，积极推进素质教育，推动各级各类教育协调发展。提高义务教育水平，加快发展学前教育，进一步规范普通高中教育，大力发展职业教育，鼓励和引导民办教育健康发展。实施幼儿园标准化建设工程，推进中小学校舍安全改造、“两热一暖一改”试点工程，加快中心城区义务教育学校建设和市技术学院迁建。全面落实家庭经济困难学生资助政策，认真解决农民工子女义务教育问题。推动医药卫生事业改革发展，健全基层医疗卫生服务体系，建设352处村卫生室、3处社区卫生服务中心和15处社区卫生服务站；从今年起，按一定标准对承担公共卫生服务职能的在岗乡村医生给予补助。大力实施基本药物制度，从今年起，全市所有政府办的城乡基层医疗卫生机构配备和使用基本药物，实行“零差率”销售。扎实推进公立医院改革试点工作。深入实施公共卫生服务项目，推进基本公共卫生服务均等化。扎实做好手足口病、甲型H1N1流感等重大疫病防控。抓紧抓好人口与计划生育工作，切实稳定低生育水平。加强国防教育，抓好民兵预备役建设和人民防空工作，巩固扩大双拥共建成果，争创新一届全国双拥模范城。大力发展残疾人事业，争创全国残疾人工作示范城市。关心支持妇女儿童和青少年事业，做好老龄工作。进一步做好外事侨务、民族宗教、无线电管理、档案、气象、对台、地名、史志、仲裁、红十字会、防震和减灾救灾等工作。

　　(三)加强和谐文化建设。以打造黄河口文化品牌为目标，以大平台、大活动、大集团为抓手，大力发展文化事业和文化产业，推进经济文化融合发展。完善公共文化服务网络，加快建设一批重点文化体育设施；加强基层文化体育场所建设，建设改造中心城社区文体活动中心5处、乡镇综合文化站34处。办好2010年黄河口国际马拉松赛。积极开展群众性体育健身活动，加快竞技体育发展。深化文化体制改革，加快推动经营性文化单位转企改制，全面推进文化市场综合执法改革和广播影视体制改革。鼓励发展文化企业，打造一批文化精品，培育一批龙头企业。繁荣发展哲学社会科学。加强社会主义核心价值体系建设，深入开展“从我做起，走向文明”主题实践活动，扎实推进创建“文明城市”工作，提高全社会文明程度。加强民主法制建设，深入开展普法教育，扩大基层民主，做好村居换届选举工作。

　　(四)切实加强社会管理。深入推进社会管理创新，理顺重点领域社会管理体制，积极构建属地管理、权责明确、规范科学的社会管理新格局。扎实推进“平安东营”建设，夯实基层基础工作，完善社会治安防控体系。深入推进以“打黑除恶”为重点的严打整治斗争，加强社会和油区治安综合治理，强化出租房屋和流动人口综合管理，配套完善居民小区物防、技防设施和保安巡防力量，创新农村治安防范模式，维护良好的社会治安秩序。完善矛盾纠纷排查化解机制，落实领导接访、干部下访等制度，着力化解突出信访问题，积极预防和妥善处理群体性事件。坚持和完善市长公开电话、市长市民对话会制度，畅通社情民意表达渠道。认真开展“安全生产基层基础年”活动，加大隐患排查力度，抓好重点行业和领域安全生产专项整治，坚决遏制重特大生产安全事故的发生。高度重视食品药品安全工作，落实监管措施，严厉打击制假售假行为。进一步完善应急管理体制机制，提高应急处置能力。

　　“十二五”是我市全面建设小康社会、加快推进黄河三角洲高效生态经济区建设的关键时期，我们要科学编制好“十二五”规划，为“十二五”的发展奠定基础。

七、切实加强政府自身建设

　　新的形势和任务，对政府自身建设提出了新的更高的要求。各级政府必须增强责任感、使命感，树立新境界，强化新举措，谋求新作为，切实肩负起历史赋予的重任。

　　(一)解放思想，提升境界。加快黄河三角洲高效生态经济区建设是一场深刻的革命，必须进一步解放思想、开拓创新，奋力打开新局面、实现新发展。要牢固树立终身学习的理念，努力建设学习型政府、学习型机关。要站在实施国家战略的高度提升思想境界，用心研究事关全局的重大问题，树立世界眼光，加强战略思维，努力做到以宽广的视野谋划全局、以非凡的气魄推动发展。切实增强改革创新意识，认真吸收借鉴先进经验，大胆探索，勇于实践，真正做到用新的发展理念指导实践，用新的体制机制解决问题，用新的方式方法推动工作。始终保持奋发有为的精神状态，自我加压，攻坚克难，瞄准一流，奋力争先，努力在克服困难中开拓前进。

　　(二)转变职能，依法行政。结合政府机构改革，优化政府组织结构，加快政府职能转变，增强政府提供公共服务的能力。深化行政审批制度改革，进一步清理和规范行政审批事项。深入推进政务公开，提高政府工作透明度。全面推进依法行政，严格按照法定权限和程序行使权力、履行职责。坚持科学民主决策，完善公众参与、专家论证和政府决策相结合的决策机制。加强行政行为的规范和监督，确保严格执法、公正执法、文明执法。严格执行人大及其常委会的决议决定，支持人民政协履行职能，认真办理人大代表建议和政协提案，主动听取各民主党派、工商联、各人民团体和人民群众的意见建议，自觉接受各方面监督。

　　(三)求真务实，真抓实干。大力弘扬求真务实精神，坚持出实招、办实事、求实效，坚决克服形式主义、官僚主义。倡导扎实深入的工作作风，深入实际，深入基层，深入群众，铺下身子抓落实，集中精力解难题。强化实践锻炼，在改革发展的主战场、维护稳定的第一线、服务群众的最前沿经受考验，历练成长，增长才干，努力提高把握大局的能力、破解难题的能力、创新发展的能力、处理复杂问题的能力和推动科学发展的能力。牢固树立全市一盘棋的思想，围绕大局，加强协调配合，提高整体战斗力。增强服务意识，提高工作效率，优化发展环境。健全完善目标责任制、工作评价机制和行政问责制，加强督查考核，严格责任追究，提高政府的执行力和公信力。

　　(四)执政为民，廉洁勤政。各级政府及其工作人员必须牢固树立执政为民理念，为人民掌好权、服好务。要大力发扬艰苦奋斗精神，坚持勤俭办一切事业，坚决反对铺张浪费。坚持廉洁从政、从严治政，加强政风行风建设，健全完善惩治和预防腐败体系。深入开展反腐倡廉教育，筑牢拒腐防变的思想道德防线。坚持用制度管权、管事、管人，健全权力运行制约和监督机制。继续加大案件查处力度，严肃查处各类违法违纪案件，依法严惩腐败分子。各级政府和各部门主要负责人是廉政建设的第一责任人，要严格执行廉洁自律各项规定，自觉作反腐倡廉的表率。

　　各位代表！油地军校都是加快黄河三角洲开发建设的重要力量，我们要继续高举团结发展的旗帜，共克时艰、共促和谐、共建家园，共同创造美好未来！

　　人民群众是力量之源，我们必须紧紧依靠人民群众，集民智、聚民心、汇民力，形成无坚不克、无往不胜的强大力量！

　　各位代表！新的征程已经开启，新的目标催人奋进。让我们更加紧密地团结在以胡锦涛同志为总书记的党中央周围，坚持以邓小平理论和“三个代表”重要思想为指导，深入贯彻落实科学发展观，在省委、省政府和市委的坚强领导下，进一步解放思想，抢抓机遇，锐意进取，真抓实干，为谱写黄河三角洲开发建设新篇章、实现富民强市新跨越而努力奋斗！
